# Obituary

**Published:** 1894-12

**Authors:** 


### Obituary.

---

DR. WILLIAM GOODELL, of Philadelphia, died in that city on October 27, 1894, in the sixty-fifth year of his age. He had been in failing health for some time, but his end was finally sudden.

Dr. Goodell was one of the well-known American physicians, and of late years has occupied prominence as a gynecologist. For twenty-two years he had charge of the Preston Retreat, during which time he was actively engaged in teaching and writing. He had remarkable literary gifts and his writings are often quoted. He was a painstaking and methodical worker and will be greatly missed, especially among the group of gynecologists of his period, few of whom are left.

